# Audiological and otologic manifestations of glutaric aciduria type I

**DOI:** 10.1186/s13023-020-01571-w

**Published:** 2020-12-01

**Authors:** Yen-Chi Chen, Chii-Yuan Huang, Yen-Ting Lee, Chia-Hung Wu, Sheng-Kai Chang, Hsiu-Lien Cheng, Po-Hsiung Chang, Dau-Ming Niu, Yen-Fu Cheng

**Affiliations:** 1 Department of Otolaryngology-Head and Neck Surgery, Taipei Veterans General Hospital, Taipei, Taiwan; 2 Department of Otolaryngology-Head and Neck Surgery, Kaoshiung Municipal Gangshan Hospital (Outsourceded by Show-Chwan Memorial Hospital), Kaoshiung, Taiwan; 3 Department of Medical Research, Taipei Veterans General Hospital, Taipei, Taiwan; 4 Department of Otolaryngology-Head and Neck Surgery, School of Medicine, National Yang-Ming University, Taipei, Taiwan; 5 Department of Pediatrics, Taipei Veterans General Hospital, Taipei, Taiwan; 6 Institute of Clinical Medicine, National Yang-Ming University, Taipei, Taiwan; 7 Department of Biomedical Engineering, National Yang-Ming University, Taipei, Taiwan; 8 Institute of Brain Science, National Yang-Ming University, Taipei, Taiwan; 9 Department of Radiology, Taipei Veterans General Hospital, Taipei, Taiwan; 10 School of Medicine, National Yang-Ming University, Taipei, Taiwan

**Keywords:** Glutaric aciduria type 1, Hereditary hearing loss, Syndromic hearing loss

## Abstract

**Background:**

Glutaric aciduria type 1 (GA-1) is a rare disease connected with speech delay and neurological deficits. However, the audiological and otologic profiles of GA-1 have not yet been fully characterized. To our knowledge, this is the largest study of comprehensive audiological and otologic evaluation in patients with GA-1 to date.

**Methods:**

Thirteen patients diagnosed with GA-1 between January 1994 and December 2019 with audiological, radiological and genetic manifestations were retrospectively analyzed. Hearing tests were performed in all patients. MRI was performed for radiological evaluation.

**Results:**

Hearing loss was found in 76.9% (10/13) of GA-1 patients, including slight hearing loss in 46.1% (6/13) of patients, mild hearing loss in 15.4% (2/13) of patients, and moderate hearing loss in 7.7% (1/13) of patients. Normal hearing thresholds were seen in 23% (3/13) of patients. Patients with intensive care unit (ICU) admission history showed significantly worse hearing than those without (29.17 ± 12.47 vs 13.56 ± 3.93 dB HL, 95% CI 2.92–24.70, *p* = 0.0176). One patient had moderate sensorineural hearing loss and a past history of acute encephalopathic crisis. No usual causative gene mutations associated with hearing loss were found in these patients. MRI showed a normal vestibulocochlear apparatus and cochlear nerve. One patient with extensive injury of the basal ganglia on MRI after acute encephalopathic crisis was found to have moderate sensorineural hearing loss. Two patients with disability scores above 5 were found to have mild to moderate hearing impairment. No obvious correlation between macrocephaly and hearing loss was found.

**Conclusion:**

A high prevalence of hearing impairment is found in GA-1 patients. Adequate audiological evaluation is essential for these patients, especially for those after encephalopathic crises or with ICU admission history.

## Introduction

Glutaric aciduria type 1 (GA-1) is a rare autosomal recessive metabolic disease first described in 1975 [1]. The worldwide prevalence of GA-1 is estimated at 1:110,000, with approximately 500 or more cases reported to date. It is caused by mutations in the *g*lutaryl-*C*oA *d*e*h*ydrogenase (GCDH) gene mapped to chromosome 19p13.2, which encodes a flavin adenine dinucleotide-dependent mitochondrial matrix protein that is involved in the degradation of l-lysine, l-hydroxylysine, and l-tryptophan [2]. The defect of the GCDH enzyme in the oxidative pathway leads to an accumulation of glutaric acid (GA), 3-hydroxyglutaric acid (3-OH-GA), glutaconic acid, and glutarylcarnitine (C5DC).

As GA and 3-OH-GA are putatively neurotoxic, GA-1 is characterized by neurological impairment, macrocephaly, dystonia and seizure [3, 4]. Without proper treatment, approximately 90% of infants will develop acute encephalopathic crisis before the age of 3, which is during a vulnerable period of brain development [5]. According to the literature reports, patients are still susceptible to acute encephalopathic crisis even under strict treatment [6]. The diagnosis of GA-1 is made based on analyzing the C5DC concentration in dried blood spots during newborn screening and confirmed by mutations in the *GCDH* gene. MRI often reveals a characteristic pattern of gray and white matter abnormalities and widened cerebrospinal fluid (CSF) spaces in GA-I. A recent study described that the severity of movement is related to extensive lesions of the putamen [7]. Typically, patients present nonspecific neurologic symptoms within their infancy. Macrocephaly is a frequent (75%) but nonspecific finding and is present at or shortly after birth. Individual speech delay and abnormal ocular findings have also been reported [8, 9]. Neurologic sequela after crisis leads to acute bilateral striatal injury and, subsequently, a complex movement disorder. Morbidity and mortality are high in these individuals after encephalopathic crisis [5]. Treatment is analogous to that for other organic acidurias, including dietary treatment in combination with oral supplementation of l-carnitine. Mortality and morbidity are considerably reduced after proper treatment, and GA-I is therefore considered to be a treatable condition.

Most children are found to have some degree of speech difficulty (e.g., dysarthria, articulation difficulties). Psychological functions including intelligence for assessment at the general level of development, motor functions and language have been suggested. GA-1 was reported to be associated with hearing loss in isolated case reports [10]; the sparse number of reports may be due to the small patient population of GA-1 or insufficient newborn screening systems. To date, no study has explored otologic and audiological profiles in patients with GA-1. Here, we describe the audiological and otologic manifestations of 13 GA-1 patients (12 by newborn screening and one by clinical diagnosis), and all of them were retrospectively evaluated at a single tertiary referral hospital. This study is the first and largest comprehensive report to describe the otologic and auditory findings in GA-1 patients.

## Methods

### Patients

From January 2001 to November 2019, all patients diagnosed by newborn screening (NBS) underwent secondary confirmatory diagnosis by including quantitative analysis of GA and 3-OH-GA in urine and/or blood and *GCDH* gene mutation analysis. Of the 16 patients who were diagnosed with GA-1 at Veterans General Hospital, 13 patients underwent comprehensive otologic evaluation and audiological tests between September 1994 and December 2019. The demographic data of the patients were retrospectively collected from their medical records, including newborn hearing screening results, age at diagnosis of GA-1, occurrence of clinical encephalopathic crisis, disability score and length of follow-up. A history of intensive care unit (ICU) admission was also recorded.

GA-1 treatment was started immediately after diagnosis. The maintenance treatment consisted of carnitine and a low lysine diet supplemented with a GA-1 special formula. Parents were given instructions regarding emergency treatment. During intercurrent illness, catabolism needs to be prevented promptly by providing a high-energy intake. Patients were seen at our clinic every 3 months. T1-weighted and T2-weighted MRI, diffusion-weighted imaging (DWI) and apparent diffusion coefficient (ADC) maps were arranged at the time of diagnosis, and new onset neurological symptoms were identified. Imaging data were not available for some patients because some parents were reluctant to subject asymptomatic children to MRI under sedation.

### Audiological tests

Pure tone audiometry or conditioned behavioral audiometry (either reinforcement audiometry or conditioned play audiometry) was arranged for hearing assessment. A sound field test was conducted if the patient was not amenable to the headphone. Auditory brainstem response (ABR) was conducted for patients with poor auditory responsiveness resulting from any cognitive-related impairment. The audiological tests were conducted in a double-wall booth by an experienced audiologist. Air-conduction pure tone audiometry thresholds were tested at frequencies of 250, 500, 1000, 2000, 4000, and 8000 Hz. The pure tone average (PTA) was determined by calculating the threshold of frequencies at 500, 1000, 2000 and 4000 Hz. Our classification of the degree of hearing loss was based on calculating the PTA. There was no hearing loss if the PTA was less than 16 dB HL, 16–25 dB HL was slight hearing loss, 26–40 dB HL was mild hearing loss, 41–55 dB HL was moderate hearing loss, 56–70 dB HL was moderately severe hearing loss, 71–90 dB HL was severe hearing loss, and ≥ 91 dB HL was profound hearing loss in accordance with the American Speech Language Hearing Association guidelines [11, 12].

Distortion product otoacoustic emissions (DPOAEs) were measured by using the Biologic Scout Sport PC-based diagnostic OAE system. The ratio of two primary tones, f2/f1, was fixed at 1.22, and moderate-level primary tones were used with L1 = 65 dB SPL and L2 = 55 dB SPL. The DPOAE measurements were over the frequency range 1–8 kHz and interpreted as present when the signal-to-noise ratio (SNR) exceeded 6 dB or more at the f2 frequency. DPOAE was interpreted by the SNR criterion. DPOAE criteria of 6-dB DPOAE/noise ratios or better determination of the presence of DPOAE and five frequencies were required to meet the criterion classified as normal hearing.

### Targeted resequencing of 4 deafness-related genes (*SLC26A4, GJB2, GJB3* and mtDNA 1555 and 1494) by next-generation sequencing (NGS)

All of the patients underwent targeted sequencing of 4 common genes related to deafness in Taiwan, including *SLC26A4*,* GJB2*,* GJB3* and mtDNA 1555 and 1494. First, we designed PCR primers for all candidate genes mentioned above by the online website tool Ion AmpliSeq™ Designer (Thermo Fisher). The expected sizes of all amplicons were set to 275 bps. A total amount of 10 ng of DNA from each individual was used to construct a sample library. All designed primers were mixed according to well-optimized proportions and used for multiplex PCR. After amplification, the reactants were purified and quantified by a Qubit fluorometer (Thermo Fisher). Proper amounts of reactants were subjected to second-stage library preparation, including adapter and barcode ligation to both ends of all amplicons, and size selection (AMPure XP, Beckman Coulter). The barcoding amplicons of each individual were analyzed by a Bioanalyzer system (Agilent) to ensure the sizes and quantities of the library products. The qualified libraries were subjected to sequencing reactions by “sequencing by synthesis” (SBS) technology on a MiSeq sequencer (Illumina). The sequencing kits with different sequencing throughputs were chosen according to the amounts of library. The average depth of the target regions was more than 200 ×. The data analysis, including quality control, reference sequence alignments, and variant annotations, was executed on the BaseSpace Sequence Hub webtool (Illumina).

### Disease severity

The disability score, which is the summation of the scores for motor function, cognitive function and speech, giving a minimum of three and a maximum of nine, was developed and found to be significantly correlated with acute onset, dystonia and mortality [13]. The disability scores were simultaneously collected when the audiological test was arranged.

### Statistics

The data are expressed as the mean, and descriptive statistics were also performed. Statistical analyses to compare the hearing thresholds of ICU admission, encephalopathic crisis, macroencephaly, and severe disability were performed using paired *t* tests for normally distributed data. Statistical significance was identified when *p* < 0.05. Calculations were performed using GraphPad Prism, Version 8 (GraphPad Software, CA, USA).

## Results

### Patients

The demographic data of the patients included in the study are shown in Table 1. Thirteen patients were enrolled in this study, including 3 male and 10 female patients, ranging from 2 to 26 years old (mean: 8.0 years old). Case 13 was diagnosed clinically due to birth before the initiation of the nation-wide newborn screening for GA-1. All patients received DNA testing to confirm *GCDH* mutations. The mean age of treatment initiation was 8.9 days from birth (excluding case 13, who started treatment at the age of 14). A total of 23.0% (3/13) of the patients experienced encephalopathic crisis and showed some degree of motor dysfunction.

**Table 1:** Characteristics and audiological results of 13 patients with GA-1

Case no	Sex	Age	Age at treatment initiation	Clinical presentation	OAE	Audiogram	Hearing grading	Newborn hearing screening	Encephalo-pathic crisis	Macro-cephaly	Disability score	ICU admission
1	F	2	8 days	Asymptomatic	P/A	SF	Mild	Pass	No	N/A	3	Yes
2	M	2	6 days	Asymptomatic	P/P	SF	Slight	pass	No	No	3	No
3	M	3	8 days	Asymptomatic	P/P	SF	Slight	Pass	Yes	Yes	3	Yes
4	F	3	8 days	Dystonia/NG feeding	A/P	ABR	Mild	Pass	Yes (4 m)	No	8	Yes
5	F	9	9 days	Seizure, developmental delay	B/P	SRT	Mild	Pass	No	No	6	No
6	F	3	8 days	Seizure	P/P	SF	Moderate	Pass	Yes	Yes	5	Yes
7	F	4	8 days	Asymptomatic	P/B	PTA	Slight	Pass	No	No	3	Yes
8	M	7	6 days	Asymptomatic		PTA	WNL	Pass	No	Yes	3	No
9	F	11	9 days	Mild developmental delay	B/B	PTA	Slight	Pass	No	Yes	4	No
10	F	16	1 months	Very mild involuntary movement and unstable gait	B/B	PTA	WNL	Pass	No	Yes	3	No
11	F	11	10 days	Optic atrophy, developmental delay	B/B	PTA	Slight	Pass	No	Yes	3	No
12	F	7	7 days	Asymptomatic	B/B	PTA	Slight	Pass	No	No	3	No
13	F	26	12 years	Asymptomatic	P/P	PTA	WNL	Nil	No	No	4	Yes

*B* broad intact, *P* partial loss, *A* absent, *SF* sound field, *PTA* pure tone audiogram, *SRT* speech recognition test, *ABR* auditory brainstem response

### Audiological and otologic findings

**Fig. 1:**
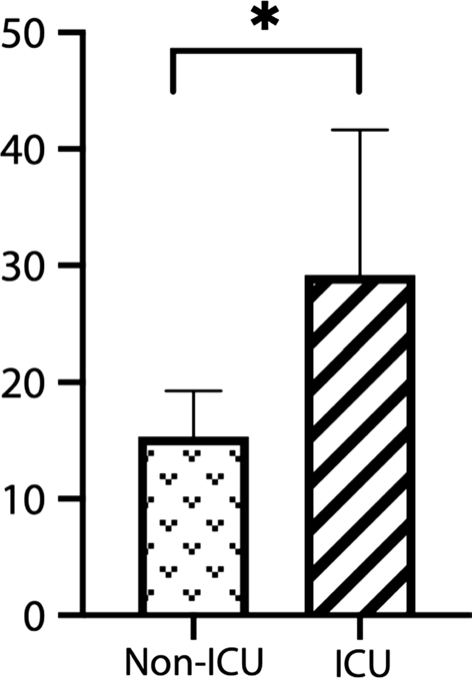
Mean hearing thresholds in patients with and without an ICU admission history. Patients with a history of ICU admission showed worse hearing performance than those without (mean ± SD = 29.17 ± 12.47 vs 13.56 ± 3.93 dB HL, 95% confidence interval (CI) − 0.04558 to 28.05, *p* = 0.0176, *p < 0.05)

The hearing assessment was conducted with pure tone audiometry in 7 (53.8%) patients and sound field tests in 5 (38.5%) patients. The mean age at the first hearing test was 8.14 years old. The detailed audiometric data are shown in Table 1. The mean AC value was 21.98 dB HL in all patients. Hearing thresholds were normal in 23% (3/13) of ears. Slight hearing loss was found in 46.1% (6/13) of patients, mild hearing loss in 15.4% (2/13) of patients, and moderate hearing loss in 7.7% (1/13) of patients. All patients diagnosed with hearing loss had sensorineural hearing loss in nature. Two patients with disability scores above 5 had mild to moderate hearing impairment. In total, 46.1% (6/13) of patients were reported as needing ICU admission, but only half of these patients (3/6) was reported to have had an encephalopathic crisis; others were admitted due to febrile illness or seizure. None of these 6 patients received ventilation, inotropic support or hemofiltration during ICU admission. Additionally, none of these patients had received known ototoxic medication such as aminoglycoside antibiotics. The confounding of patients with ICU admission is listed in Table 2. Patients with and without ICU admission showed a significant difference in hearing loss (29.17 ± 12.47 vs 13.56 ± 3.93 dB HL, 95% confidence interval (CI) 2.92–24.70, *p* = 0.0176, Fig. 1), indicating worse hearing performance in patients with a history of ICU admission. A history of acute encephalopathic crisis had a negative impact on hearing performance but no statistical significance (32.50 ± 14.09 vs 18.50 ± 8.412 dB HL, 95% confidence interval (CI) − 0.04558 to 28.05, *p* = 0.0506). No obvious correlation between macrocephaly and hearing loss was found (*p* = 0.977). No otological anomalies, such as atresia of the auricle or external ear canal, otitis media with effusion or any infection or inflammatory sources, were found in these patients. DPOAEs from 12 patients showed absent or partial loss in 62.5% (15/24) of ears (Table 1), and 11 of these patients showed slight to moderate hearing loss. The OAE results seemed highly compatible with the PTA findings.

**Table 2:** MRI characteristics of the 13 patients

Case no	Patient’s age at imaging	Widened Sylvian fissures	Hyperintense T2 caudate/putamen	Presence of pseudocyst	Restricted diffusion in basal ganglia	Subdural hematoma or hygroma	Detectable lesion along the auditory pathway
3	1 year	+	+	−	+	−	−
5	12 days	+	+	−	+	−	−
6	1 year	+	+	−	+	−	−
7	18 days	+	−	−	−	−	−
8	24 days	+	+	−	−	−	−
9	10 days	+	−	−	−	−	−
10	8 years	+	−	+	+	−	−
11	4 months	+	−	−	−	−	−
12	24 days	+	−	−	+	−	−
13	1 year	+	+	−	+	−	−

The imaging characteristics of the 13 patients enrolled in this study are listed. In cases 1, 2 and 4, imaging data were not available in our hospital. The findings in this table were identified by MRI upon diagnosis

### Genetic testing findings

Genetic testing confirmed *GCDH* mutations in all enrolled patients. For the four deafness-related genes tested, including *SLC26A4, GJB2, GJB3* and mtDNA 1555 and 1494, no pathological variants were found, indicating the origin of hearing loss from the pathological nature of GA-1.

### Radiological findings

**Fig. 2:**
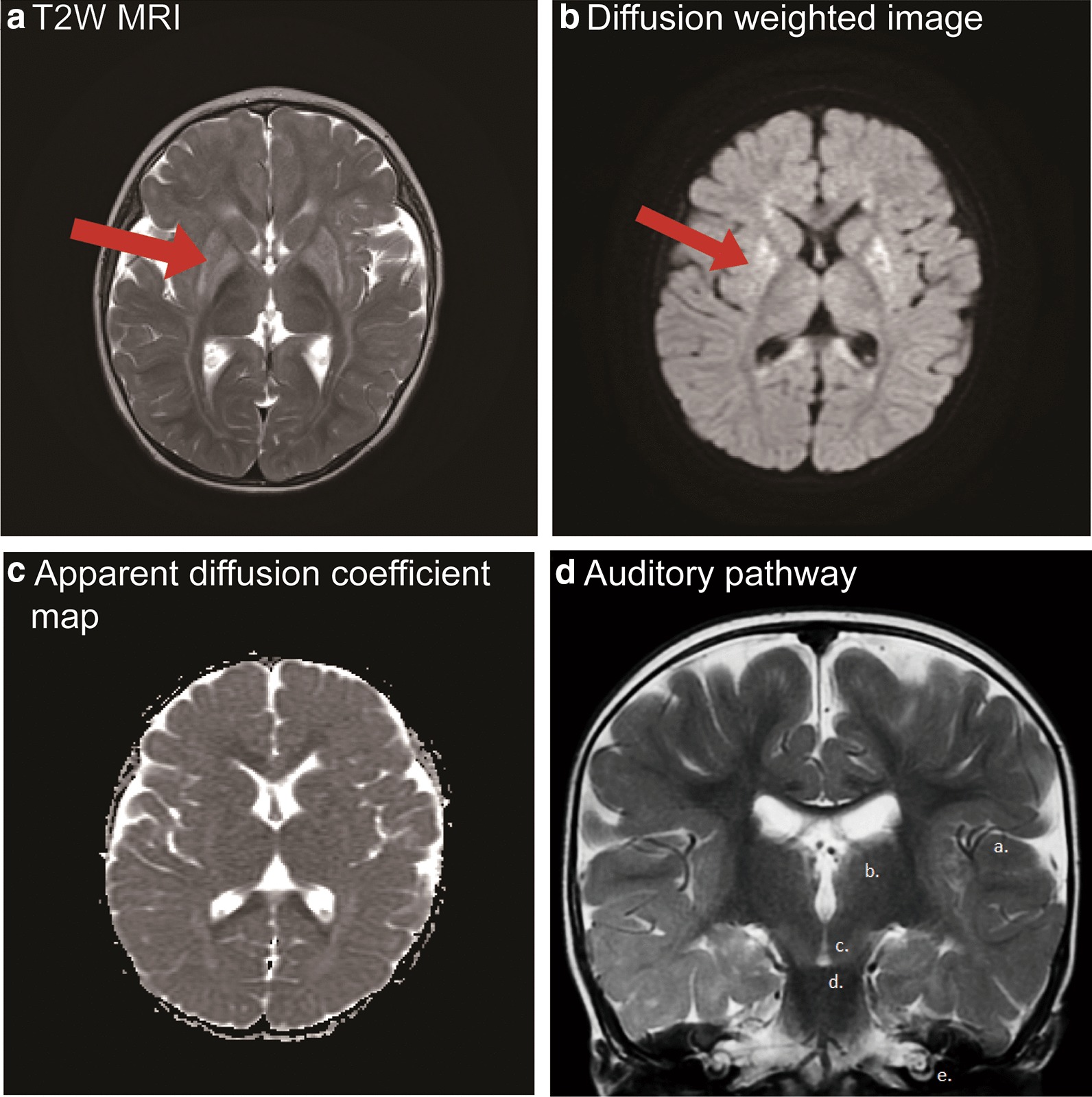
MR images of patients with GA-1. A-C. The pattern of putamen injury in case 6 with a history of seizure and acute encephalopathic crisis is shown. (**A**) T2 weighted (T2W) MRI. The red arrow indicates extensively hyperintense striatal involvement in T2W MRI. (**B**) Diffusion weighted images. The red arrow reveals injury with clearly elevated diffusion of the dorsolateral putamen on diffusion weighted images. (**C**) Apparent diffusion coefficient map. The image shows minimal restricted diffusion affecting the bilateral putamen. (**D**) Illustration of imaging of the central auditory pathway. An oblique coronal section onT2WI MRI of case 3 shows the hypointense white matter tract. Letters (a) to (e) denote the apparatus of the auditory pathway. (a) Auditory cortex, (b) Thalamus (medial geniculate body), (c) Midbrain (inferior colliculus), (d) Pons (superior olivary nucleus), (e) Cochlea. Note that no space occupying lesion was noted along the tracts

To explore the possible involvement of the auditory pathway, MRI investigations were evaluated in all thirteen patients. There was no vestibulocochlear malformation, atresia of the internal acoustic canal (< 4 mm), or absent or hypoplastic cochlear nerve in these patients. Furthermore, no lesions were noted along the central auditory pathway, including the cochlear nucleus (CN), superior olivary nucleus (SON), inferior colliculus (IC), medical geniculate body and auditory cortex. One of the patients (case 6) showed extensive putamen lesions on MRI (Fig. 2a–c). An oblique coronal section on T2-weighted imaging (T2WI) of patient 3 is shown as an example of comprehensive auditory pathway evaluation (Fig. 2D). The clinical presentation of the patient revealed moderate hearing loss, history of seizure attack, and impaired speech and motor functions. Table 2 shows a summary table of the MRI results and key findings for all patients.

## Discussion

GA-1 is a progressive disorder that typically appears normal at birth. However, patients may experience various degrees of permanent neurological sequelae, especially after encephalopathic crisis, including motor and cognitive dysfunctions and mental retardation. Newborn screening of GA-1 started in 2001 and has been publicly offered by the National Health Insurance system in Taiwan since 2006. Our previous data revealed that the incidence of GA-1 was 1 in 106,474, which is approximately the same worldwide and in the Taiwanese population [4, 14]. To our knowledge, this is the first study to investigate the audiological and otologic profiles of GA-1. Our study may enhance awareness of early intervention in these children before dysarthria and other hearing loss-related morbidities occur (Table 3).

**Table 3:** Characteristics of GA-1 patients admitted to the ICU

Case no	Sex	Age at diagnosis	Age at ICU admission	Cause of admission	Ventilation	Inotropic support	Hemo-filtration	Ototoxic antibiotics	Disability score before ICU admission	Disability score after ICU admission
2	F	2	1 year	Febrile seizure	Nil	Nil	Nil	Nil	3	3
4	M	3	1 year	AGE (which then progressed to AEC)	Nil	Nil	Nil	Nil	3	3
5	F	3	5 months	AEC	Nil	Nil	Nil	Nil	3	8
7	F	3	4 months	AEC	Nil	Nil	Nil	Nil	3	5
8	F	4	8 months	Febrile illness (> 40 °C)	Nil	N/A	nil	N/A	3	3
16	F	26	1 year	Febrile seizure	Nil	N/A	Nil	N/A	3	4

*AEC* acute encephalopathic crisis, *AGE* acute gastroenteritis

We found that a high percent of patients with GA-1 encountered slight to moderate sensorineural hearing loss in this study. The metabolite of GA-1 is neurotoxic, and accumulation in the central nervous system can cause severe neurological complications. The presence of 3-OH-GA in GA-1 patients may lead to increased vulnerability of endothelial structures and subsequent vascular dysfunction. The cochlea is the end organ fed solely by vessels of the labyrinthine artery, making it vulnerable to circulation causes [15]. Like other toxic metabolic diseases affecting the auditory system, the pathophysiology of hearing loss is still unclear [16]. However, it is anticipated that oxidase and neurotoxic substance accumulation in the cochlea or the rest of the auditory system causes hearing loss [17]. Other metabolic diseases such as Mitochondrial Encephalopathy, Lactic Acidosis, and Stroke-like episodes (MELAS), homocystinuria, mucopolysaccharidosis and congenital hyperlactic acidemia were also reported directly or indirectly related to hearing loss [18–23]. Several lysosomal storage disorders have been reported to affect hearing functions in the pediatric population. For example, in classic Fabry disease patients, it was postulated that globotriaosylsphingosine (lyso-Gb3), a neurotoxic substance predominantly deposited in these patients, causes stria vascularis injury and spiral ligament atrophy [24]. In the animal model of Pompe disease, glycogen accumulation was found in the cochlea, affecting inner and outer cells and finally leading to hearing loss [25]. An animal model of GA-1 (*Gcdh*^−/−^ mice), demonstrated typical motor deficits and diffuse spongiform myelinopathy, but did not seem to show any hearing phenotypes [26]. While hearing loss is suspected to be caused by the accumulation of toxic metabolites in the auditory pathway, including the cochlea, auditory nerve, and brainstem, further studies are needed to elucidate the causes.

We found that 75% of GA-1 patients had varying degrees of hearing loss in our study. The high prevalence of hearing loss was not reported previously. OAEs showed partial missing signals, indicating partial loss of function of the outer hair cells in the cochlea. It has been shown that over 50% of prelingual hearing loss has attributable genetic factors [27]. To preclude other possible genetic causes of hearing loss, the four most common deafness-associated mutations in the Taiwanese population, *GJB2*, *SLC26A4*, *GJB3,* and mitochondrial *m.1555A* > *G*, were included in our NGS panel. In terms of the allele frequency in the hearing-impaired population, these 4 mutations account for over 80% of the known deafness-associated mutations in Taiwan [28]. We did not find any pathologic variants of the four genes identified in our study. The inclusion of the 4 common deafness-associated genes in the genetic tests offers strong evidence to preclude hearing loss caused by the popular deafness-related gene variants in the Taiwanese population.

MRI offers the advantage of detecting causes of sensorineural hearing loss, including large vestibular aqueduct, inner ear anomaly, cochlear nerve deficiency and postinfection changes [29]. In our study, all MRI series showed negative findings of the peripheral auditory apparatus, including the vestibulocochlear apparatus, cochlear nerve and endolymphatic duct, or the central auditory pathway. As such, the hearing impairment of children with GA-1 was not structural or caused by infections related to the peripheral or central auditory organs. In addition, the DWI and ADC maps have value for differentiating cytotoxic brain injury from vasogenic brain edema [30]. We found that most of the brain edema in our patients was cytotoxic edema. It was postulated that 76% of patients with GA-1 and 88% after encephalopathic crisis have injury over the putamen on MRI [31]. We found that one patient with acute encephalopathic crisis, who had moderate sensorineural hearing loss, had extensive injury of the basal ganglia on MRI. The relationship between hearing loss and cytotoxic brain injury requires further clarification in the future.

The evolution of diffusion tensor imaging in MRI has led to the finding of an association between sensorineural hearing loss and white matter tract microstructure [32]. Some extensive functional MR sequences, including MR spectroscopy, have been proposed to examine patients with GA-1 [33]. Fractional anisotropy (FA) is widely used as an important parameter to change the functional integrity of white matter. The central auditory pathway, including the auditory cortex and IC, was found to have significantly lower FA values than normal hearing subjects, which is widely used as an important parameter to determine the directionality of diffusion [34]. However, most parents are reluctant to allow the patients receive these functional sequences because the prolonged general anesthetic duration in pediatric patients may increase the risk of neurodevelopmental effects [35]. It has also been reported that the microstructures of the brain have clinical value in the determination of speech and language therapy [36]. It is of interest to further determine the structural or functional changes within the auditory pathway among GA-1 patients with hearing impairment in the future.

In our study, hearing loss was found in 69.2% of patients, which is much higher than the global prevalence of pediatric hearing impairment (7.6–16.4% worldwide [37–39]). However, all of our patients passed the newborn hearing screening, implying that onset hearing loss may not occur at birth but instead during later childhood. It is well-recognized that hearing is critical for speech development in the early years of a child’s life, and even mild sensorineural hearing loss may also cause a negative impact on the academic performance or language skills of children [40–43]. GA-1 was reported to affect fine motor skills and speech, and early intervention and rehabilitation were suggested to optimize the educational environment for patients with GA-1 [6, 44, 45]. As there is no report regarding the audiological and otologic manifestation of GA-1, a routine hearing test is not included in the consensus guidelines [6]. Based on this study, we suggest routine audiological and speech screening after birth for GA-1 patients.

Some risk factors have been reported to affect hearing performance, such as ICU admission, ototoxic drug use, genetic syndromes and recurrent middle ear infection [38, 46]. In our study, hearing loss was found in 5 of 6 patients with a history of ICU admission. In addition, a significantly higher average hearing threshold was also found in patients with a history of ICU admission. Previous studies showed that the prevalence of hearing loss in the ICU population was 3.2%, which is significantly lower than our result [47, 48]. While several factors have been reported to be associated with poor hearing in patients with ICU admittance, such as sepsis, meningitis, cerebral bleeding, antibiotic use and noise exposure [48, 49], further studies are needed to explore the underlying reasons for poor hearing performance in those with a history of ICU admission.

Although the results of the present study showed a high prevalence of hearing impairment among these children, there were several limitations, such as the nature of retrospective and cross-sectional observational studies, the small number of patients, and the use of a single medical facility. Therefore, data on other predisposing factors leading to hearing loss could not be fully collected, such as the use of ototoxic medications, noise exposure, infections during pregnancy and trauma. Furthermore, the origins of sensorineural hearing loss in patients with GA-1 also require further neuropathological and/or radiological studies. Nevertheless, this study provides the first evidence that children with GA-1 have a high prevalence of abnormal hearing performance, and further studies are needed to address rehabilitative and educational issues.

## Conclusion

Hearing loss is a common issue in patients with GA-1. Clinicians should consider the high prevalence of hearing loss in these patients, especially in patients with encephalopathic crisis or ICU admittance. Further studies are needed to investigate the nature of hearing impairment in GA-1.

## Data Availability

Not applicable.

## Abbreviations

GA-1: Glutaric aciduria type 1
PTA: Pure tone audiometry
DPOAE: Distortion product otoacoustic emissions
ABR: Auditory brainstem response
NBS: Newborn screening
